# Book Reviews

**Published:** 1808-08

**Authors:** 


### CRITICAL ANALYSIS
### OF THE
### RECENT PUBLICATIONS
### ON THE
### DIFFERENT BRANCHES OF PHYSIC, SURGERY, AND MEDICAL PHILOSOPHY.

### *Dr. Jackson, on Cold Affusion in Fevers.*

[ Continued from our last, pp. 81—89. ]

The third part begins by an examination of Dr. Currie's Theory of Fever, and effects of the plan of treatment upon it. In this, much candour, much diligence, and great acuteness are evinced; but we fear the detail which Dr. J. seems to conceive impartiality obliged him to give with so much minuteness, will prove tedious to the common reader. It is hardly necessary to remark, that the principal causes of fever assigned by Dr. Currie, remote and proximate, are, as by most other writers, referred to debility, spasm, increased action, increased heat, and the association of each with the other.

Dr. Jackson next offers a statement of his own views of fever; which, without pretending to mark all the successive changes which take place, and which vary under different circumstances, according to the state of the subject and the nature of the impression, he calls an alteration in the organic actions of the system. That to describe, or even to define, fever, it is not enough to say, generally, that these actions are altered. It is necessary to mark the form of those alterations in all their varieties, and the effect of remedies on each. That to do this, a minutenes of description and accuracy of detail is necessary, beyond what can be interesting, or, perhaps, always intelligible to the common reader. On this account a publication, which professes almost a single remedy, and renders the application of that remedy simple by the facility with which the rules and the exceptions are understood, may be very properly called popular, inasmuch as whatever it teaches is within the scope of the uninstructed people. Such appears to be the object of Dr. Currie's Reports, which may, with still more propriety, be called popular, when contrasted with Dr. Jackson's, the minuteness of whose descriptions, added to the boldness of his practice, requires a habit of observation which can only be expected in those who are accustomed, or even devote their lives, to the practice of medicine.

Some pains are taken to connect the cause of fever with its cure. A short outline follows of the difference in the form of action according to the cause; and, in considering the treatment, the author begins with what he calls the preparation of subject. By this he means the reducing the subject to such a condition as may render render him fit for, and susceptible of, the means which are necessary to alter those wrong actions which must, if uninterrupted, end in death, or a long protracted illness and slow recovery. Among these he considers bleeding as the most important, whenever there appear marks of local congestion, imflammation, or that sluggish and torpid action which marks incapacity in the circulating vessels. Various authorities are given, to show the advantage of very free bleeding. Emetics and purgatives are advised for the same purpose. All these are considered under certain circumstances, as preparatory to the great remedy of cold affusion.—"The adjustment of the balance of action and re-action, which is held to be the base on which remedies act, is effected at one time by the operation of such means as diminish the quantity of the circulating fluids, either directly from the vessels which circulate red blood, or through some one of the emunctories which secrete and discharge superfluities; it is effected at another time, by the operation of means which solicit an equal distribution of blood and vital energy in the extreme parts; and which thus equalize the circulation of the mass, but which do not directly and evidently diminish its quantity. "I shall endeavour, says Dr. J. to describe these different conditions with as much precision as I can, aware at the sametime that I shall be held to be tedious and irksome by many; instructive and correct, perhaps, only by a few.

"In proceeding to consider this part of the subject, I shall first notice the mode of management to be adopted in that form of fever which arises from a source of personal infection, and which is now usually distinguished by the name of *typhus*. It is, perhaps, the form of fever to which cold water may be applied with greater expectations of success than any other—without previous preparation of condition by means of art. It seems to arise from sources differently concentrated, or differently modified; and, though I may not be correct, I shall state what occurs to me on this head. 1. There exists a febrile indisposition, which seems to draw its origin from a cause which is generated in crowded and ill-ventilated apartments—among persons who are not generally confined to the sick-bed. This happens frequently in jails, in ill-constructed and crowded barracks. The disease, so produced, is usually of short duration, its period rarely exceeding five days. It then subsides; but it often recurs again at a short interval. It is the least fixed of any of the forms of the infectious fever; and it yields more certainly than any of the others to the affusion of cold water on the surface, particularly when the affusion is preceded by an emetic. 2. A form of infectious fever, the cause of which is apparently referred to emanations from diseased subjects, also presents itself frequently to observation. This form arises chiefly among those who attend upon sick in hospitals, or elsewhere. Its period is longer than that of the preceding; for it rarely reaches its termination before the seventh day. It then sometimes only changes form, and it frequently passes through another septenary revolu-

 tion, tion, even sometimes two other septenary revolutions, before it terminates finally. The affusion of cold water on the surface is here ordinarily a powerful remedy in itself; but it is less sovereign than in the preceding; for, as the diseased action is strong, the mode determined and steady, the remedy does not always make impression sufficiently forcible to arrest the course abruptly and effectively—without the aid of such previous preparation as moderates the violence of the morbid action, or, as simplifies complication where that exists. 3. Infectious fever likewise presents itself, as arising apparently from a cause lodged upon clothing and such other dead matters as have been in contact with the bodies of sick persons. The cause may here be supposed to be condensed, or otherwise modified by the medium through which it is conveyed; for the effect produced actually appears with a different aspect from that of the two preceding. The movement of the morbid action is often slow and obscure; but though obscure, it is not easily acted upon, so as to be dissevered by the impression of feeble powers. The course is tedious: changes generally occur at septenary periods, but the sum of the total duration frequently extends to several weeks; and in fact, the disease often assumes one or other of the forms in which the affusion of cold water on the surface does not produce abrupt and decisive effect, unless the circumstances have been changed previously by the preparation of the fit condition.—The conditions, which I have thus described, occur to me as connected with the particular form and concentration of cause; but I am ready to confess, at the same time, that their real boundaries may not be found to be so precise in all cases as I have stated them to be. It is however true, and it must have been observed by others as well as myself, that the duration of the disease is short where the source is chiefly impure air, especially as produced by the undue accumulation of persons in ill-ventilated apartments, that is, by emanations proceeding from persons, who, though they may not be in perfect health, do not yet give evidence of the existence of actual fever. The effect of the cold affusion, as mentioned before, is here effectual, — more effectual than in the others:—preparation of subject is moreover rarely required to precede its application. The duration is long, where febrile emanation from the living body is the cause solely, or in conjunction with impure air. It is perhaps still longer, where the disease arises from the contact of impure matter alone. This is supposed to be the case with those who touch infected clothing, or who come within the sphere of condensed fomes; for persons so exposed are ordinarily observed to experience a tedious disease, and one that is comparatively little tractable to the effect of usual remedies."

The subject is further pursued under a more minute examination of the various forms of infectious fever; after which the author directs our attention to endemic fevers, or fevers arising from soil. These are divided into the mild and more simple—the more violent and complicated—the tremulous or irritated—

and, and, lastly, the stagnated or gangrenous form. The periods are next attended to, and divided into three, as the most marked.

The next subject considered is those fevers usually called epidemic. These are divided into intermittent, remittent, low autumnal, bilious fevers, dysenteric, fevers with local affections, inflammatory and eruptive fevers. On all, excepting the last, Dr. J. is as minute as usual. On eruptive fevers, such as small-pox and the other exanthemata, he professes to have seen little, but that little is favorable to cold affusion; though no expectation is indulged that in these fevers the disease can be cut short, as was often accomplished in the former. In the measles the author never conceived himself authorized to make the experiment. A recapitulation follows of the leading circumstances in which Dr. Jackson differs from Dr. Currie, the greater part of which will be unnecessary to those who peruse the previous passages in the work itself with the accuracy they deserve; and to others, we fear, it will answer but little purpose to have dwelt so long on the controversy.

The history of cold affusion is next traced from Hippocrates to the present day; and the principles on which it is used by the author are again adverted to. In detailing the rules and reasons for the application of cold water to the surface, many ingenious arguments are adduced, to show that, besides the abstraction of heat, there appears something in the external application of water peculiarly favorable to animal and vegetable life. This is illustrated in a variety of pointed instances, many of which are constantly occurring in the common transactions of life and of military duty.

Having thus gone through the principal objects of controversy, and explained most minutely the plan of operation in the affusion of cold water in fevers, Dr. Jackson directs our attention to the effects of cold drink, and of immersion in cold water, under various circumstances of disease, or of artificially excited heat. The effects of the custom are, as in the former instance, traced through the writings of the ancient and modern authors, as well medical as historical, till we arrive at the following account of the author's experience in himself.

"In the year 1779, at Savannah, in the province of Georgia, in North America, in the excessively hot weather of the month of July, I was attacked with the fever which then prevailed at that place,—a disease which in the cooler season is intermittent, but which, in the hotter months of the year, has rarely either cold stage or distinct intermission. In the third exacerbation of the case alluded to, my desire for cold water was ravenous; in short, it appeared as if the whole force of the febrile cause was exerted in the production of inordinate thirst, for there was not at the time any other symptoms which attracted my notice. In this state of torment from raging thirst, I desired to drink cold water; and, notwithstanding the unwillingness of those who were about me to comply with my request, a jug was filled from the pump,—to the best of my recollection nearly a quart. This I drank at one draught; but the thirst still continued. The draught was repeated in a few minutes—but not to the same extent: the thirst was then completely extinguished, and I could not after that well say what other part of fever remained. I went to bed about the usual time, for it was then evening, and passed the night in a pleasing reverie, or slight delirium. Next morning, when the impression of light was sufficiently strong to fix my wandering ideas, I began to examine my situation; and, among other things, found the epigastric region to be much distended, but without pain, the eye slightly yellow; while, together with this, I felt unusually languid and feeble; though I perceived no remains of fever; and even these symptoms vanished in a few days, quickened in their retreat, perhaps, by some doses of calomel and rhubarb. Such is the fact; I notice it as an instance of extinguishing fever by copious draughts of cold water; but I do not notice it as an example for the imitation of the practitioner.—The experiment was made at random; and, that no evil followed from it, was probably more owing to the original soundness of my constitution than to my caution. I do not know the precise temperature of the water employed upon this occasion; but, as it was pump-water, and drank at the instant it was drawn from the pump, it may be supposed to have been somewhere about 57°: the quantity I conclude to have been near three pints.—Such was the practice, and such were the effects in my own case. It is worthy of remark that, though the fever was extinguished in the manner stated, neither vomiting, sweating, nor any other sensible evacuation ensued,—effects which are said by writers to be the usual concomitants of water drunk in the circumstances described, and in the quantity here implied. There is here some difference in point of fact, the reason of which probably is that the ancients, and those who imitate them, generally administered the cold drink only in the advanced stage of fever, frequently near a critical period, or at the height of the paroxysm—a time, at which it is not possible to ascertain what part of the effect belongs directly to the genuine operation of the remedy, what contingently to the prepared condition of the subject. The cold water was here drunk at an early period of the paroxysm: it decidedly arrested its course or changed its condition; but I cannot, in looking back upon the case, pretend to say that it did not bring life into danger. The urgency of the thirst was unusual, and the means employed to extinguish it were carried to an unusual extent. They touched the extremes, consequently the example is not a safe one for imitation. I shall, therefore, mention, from experience in my own person also, an instance of the free use of cold water in another shape,—in such a one indeed as may be supposed to come more within the rule of common practice than that related above. In the year 1778, in the month of October, I was attacked at Kingsbridge, on New-York Island, with an intermitting fever of complicated form, attended, moreover, with some untoward symptoms through the whole whole of its course. I took an emetic at the first accession, and afterwards a strong dose of jalap and calomel; but the type, notwithstanding the effective evacutions thereby procured, still continued uncertain and anomalous, affording no fair opportunity for the exhibition of bark. I had an utter abhorrence of every sort of food or sustenance except cold water. This I drank without measure, probably not less in quantity than two gallons *per* day. The water was excellent; and, as brought directly from a spring in a rock close to my tent, it was cool and refreshing. The pitcher stood by me:—I drank whenever I had a desire to drink, without limiting myself in quantity, and I subsisted on water alone till the seventh day, when the fever terminated of its own accord. I still recollect the grateful refreshment which it gave me; and I may add, that though the fever was not extinguished by it, nor any of its paroxysms suppressed in the manner stated above, yet, the exacerbations were evidently mitigated, and I experienced comfort and even pleasure from it, not obtainable by any other means within my command at the time."

After this account of the safety and success with which the drinking of cold water was attended in himself, Dr. Jackson thinks it right to subjoin a number of cautions; so many, indeed, as may be sufficient to deter most young practitioners from the exhibition of the remedy on any terms.

The subject of cold immersion is introduced by an account of its effects on our author himself. These were such as we have seen in some other highly sensitive subjects, a high excitement of all the animal functions bordering on fever. The case of Alexander the Great, as stated by Dr. Currie, is shown to be erroneous in the main point. Dr. Currie supposes him to have been cooled as well as debilitated; but it appears, on the evidence of Q. Curtius and Arrian, that he was extremely hot when he immersed into the river Cydnus, and suffered all those inconveniencies from his rashness, which common opinion would have taught him to expect. In this instance, we should say, Dr. Currie's eager pursuit of his favorite theory, has hurried him into an unaccountable error, did we not so frequently see similar instances in many otherwise correct writers.

Some useful remarks are subjoined by Dr. Jackson, but as they rather arise from reasoning than observation, we shall omit them to take notice of a practice of which he may be said to be the author; this is, the effect of gestation in the open air in wheel carriages or other conveyances, employed as a cure for certain conditions of fever. This remedy, like many others, was suggested by chance, and in two instances on the author's own person, before he was sufficiently aware of the advantage which might be derived from it.

"In the following year, says he, I was again attacked at Ebenezer in the province of Georgia, North America, in the month of July, with fever of unusual violence. The disease was of the form which the ancient Greek physicians denominate *causus*. It had scarcely any remission, though it was fundamentally of the remitting type: the anxieties at the præcordia were inexpressible; the distress scarcely supportable; the sensation of internal heat was great; the external heat little, if in any degree increased; the abdomen was colapsed and lank; the pulsation of the descending aorta strong and vibrating; the pulsation at the wrist moderate in force, perhaps weak, and not much more frequent than natural; the tongue was parched and stiff; and together with this, there was an abhorrence of drink, which appeared nauseous and loathsome. The sensations were uncomfortable, the sense of burning was tormenting; yet the surface was frequently damp, and, as judged by the touch, not hot. The desire for something moist and cool was urgent; but nothing cool was to be met with, the thermometer rarely sinking under 96° at any time of the day in the best shaded part of the house where I lay. If my foot, or any part of my body, came in contact with any thing of wool or cotton, the irritation thus produced amounted to torture: the distress was great, and with all this there was total want of sleep, a constant desire of changing place and posture—from undescribable irksomeness. In the state described, and at the 7th day of the disease, I was put, by my own desire, into an open cart, or chair as it is termed in North America, to be conveyed to Savannah, a distance of twenty-five miles. The distress and suffering were at this time at the highest pitch possible; but I cannot pretend to give an accurate idea of it in words. It comprehended the essence of torture—without local pain. I commenced my journey in the state described; but I had not travelled two miles before my uneasines had sensibly diminished; the objects around me began to attract my notice, and I began to feel pleasure in myself. It rained heavily while I was on the road; I had no protection from it, and I was as completely drenched by it as if I had been thrown into a river; but before I reached the end of my journey, I considered myself as comparatively well. I was able to sit up, to walk without help; and, when I arrived at Savannah, I had a desire to eat something salt, though for the seven preceding days I had looked at food with abhorrence, and loathed drink with a tongue stiff and parched—even scorched to insensibility by internal heat."

A more general example of the good effect of this practice occurred in the year 1780, in the 71st Regiment, in which fever in all its stages was present. The sickness was general, and the character alarming. No less than two-thirds of the regiment were in the list. At this time the enemy approached in force, and the 71st being the advanced corps, was ordered to withdraw. Some were embarked in boats, in order to be conveyed to George Town by water. It may be supposed that the least serviceable subjects, and probable the most dangerous cases were selected for this mode of conveyance. These fell into the hands of the enemy; but of about one hundred and twenty who were placed in open waggons, illill-accommodated, exposed to night dews, scorching suns, and occasional showers, the majority were recovered or recovering by the end of the 3d day. As little or no attention could be paid to medicine during such a march, the improved health can be only imputed to the mode of gestation. A great proportion of the sick recovered whilst waiting at the first halt to take a defensive posture; others, in whom the disease had not entirely ceased, might be said to be convalescent, the form having changed from obscure remittent to that of distinct ague and fever.

The same advantages were found in the campaign in Holland, in the years 1794 and 1795, whenever it was necessary to shift the regimental hospitals and convey the sick in open carriages. In the Cove of Cork also, and in St. Domingo, the same advantages were experienced. This remedy, however, though so highly and generally valuable, is not recommended as universal. The author gives several rules for conducting it; the principal of which is, that it should be resorted to in the latter stages of fever, after natural or artificial evacuations, and where the strength seems greatly impaired.

Thus have we gone through this valuable work, which, though it contains much more useful matter than most that come before us in a much larger form; yet we are obliged to confess, might still be shortened. As this is the only objection we have to make, it is hardly necessary to add how much we recommend it to the perusal of our readers, not doubting that, like us, they will forgive this only error of the author, if they agree with us in thinking it such.

---

### *The Edinburgh Medical and Surgical Journal, No. XV.*

ARTICLE 1.—*Observations on a peculiar Affection of the Testis, attended with the Growth of Fungus from that Organ; illustrated with Cases.* By WILLIAM LAWRENCE, Demonstrator of Anatomy at St. Bartholomew's Hospital.

THIS is a very candid and instructive paper. The disease, as the author observes, is by no means rare, and has too often alarmed the surgeon in such a manner, as to induce him to propose extirpation. We shall give the description in Mr. Lawrence's own words, with an additional remark or two of our own.

"The patient has generally assigned some blow, or other injury, as the cause of the complaint; in other instances, it has originated in the hernia humoralis from gonorrhœa, and sometimes has appeared spontaneously. A painful swelling of the gland, particularly characterized by its hardness, is the first appearance of the disease. After a certain length of time, the scrotum, growing gradually thinner, ulcerates; but the opening which is thus formed, instead of discharging matter, gives issue to a firm and generally insensible fungus. The surrounding integuments and cellular substance are thickened and indurated by the complaint, so that there appears to be altogether a considerable mass of of disease. The pain abates, and the swelling subsides considerably, when the scrotum has given way. In this state, the disorder appears very indolent; but, if the fungus be destroyed by any means, the integuments come together, and a cicatrix ensues, which is inseparably connected to the testicle.

"An examination of the part, while the fungus still remains, discloses to us the fact, that this growth has its origin in the glandular substance of the testis itself; that the coats of the part are destroyed to a certain extent; and that a protrusion of the tubuli seminiferi takes place through the aperture thus formed. I have often ascertained the continuity of the excrescences with the pulpy substance of the testis; of which we shall find more or less remaining, according to the difference in the period of the disorder. It appears to me, that the glandular part of the testis experiences an inflammatory affection in the first instance, in consequence of the violence inflicted on it; and that the confinement of the swollen substance by the dense and unyielding tunica albuginea sufficiently explains the peculiar hardness of the tumour, and the pain which is always attendant on this stage of the disorder. The absorption of the coats of the testis, and of the scrotum, obviates the tension of the parts, and thereby restores ease to the patient, at the same time that the fungus makes its appearance externally."

The only objection we have to this account is, that in the cases which have occurred to us, we have had reason to suspect suppuration had taken place from the previous inflammation in the substance of the testicle. If a testicle suppurates, the consequence will be either an absorption of the matter formed, in which case, the whole testicle will appear to waste, or else the matter must find its way to the surface. In the latter case, the progress of the matter will be preceded by adhesion of the tunica albuginea to the tunica vaginalis, of the latter to the scrotum, and a subsequent absorption of all the parts between the matter and the cuticle of the scrotum. The progress is often very slow, and after the first inflammatory symptoms have ceased, very little attended to. The consequence of this slowness, added to the different functions and texture of the parts, often is an indolence or indisposition to cicatrization. Hence the granulations continue to spring up from the suppurated testicle, which prevents the aperture in the scrotum from closing. But still the attempt is made. Hence, a pressure on the sides of the granulations, which, either by consolidation of the vessels, or by a necessity of protecting themselves, produces an unusual hardness in the fungus." Mr. Lawrence continues,

"I think it not improbable, that, if the complaint were left entirely to itself, the swelling would subside, the fungus shrink, and a complete cure ensue, without any professional assistance; but the disorder is so indolent in this stage, that a spontaneous cure would not be effected until after a very long time. The excrescence may, however, be removed by the knife, or, if the nature ture of its attachment permit, by ligature; or it may be freely treated with escharotic applications. The removal of the protuberance to a level with the scrotum, by means of the knife, is the shortest and most effectual mode of treatment. I can see no ground whatever for proposing castration in this malady, since in no part of its progress, nor in any of its possible consequences or effects, can it expose the patient to the slightest risk. Some may be disposed to defend the removal of the part, because it would bring the case to a more speedy termination; and if a patient, after being informed of all the circumstances, should desire the operation on this ground, the surgeon would be justified in performing it. But I think he never could be warranted in proposing so painful and dangerous a remedy as castration for a disorder attended with neither pain nor danger, and admitting of cure by a perfectly safe and mild kind of treatment."

After this, some of his readers will be surprized that he should mention, without reserve, the name of the Gentleman who actually performed the operation under the circumstances described by himself. This is, however, a mere inadvertence; but, we are much inclined to differ from the general conclusion drawn by Mr. Lawrence in the last transcribed paragraph. When a part is so far restored, and this new situation is become chronic, it rarely happens that any spontaneous effort is made towards cicatrization. The fungus will sometimes skin over, but unless a new action is excited, or unless the fungus is kept down till the scrotum can cicatrize, the disease, if it can be called such, we have reason to believe will remain. On this occasion, may it not be reasonable to ask, whether, what is called hernia of the brain, does not arise from a similar cause, that is, from the different offices and textures of the parts concerned? And if so, does not this explain how the disease is cured by retaining the scalp, and, if possible, inducing an union of its divided edges.

We are by no means disposed to urge, that the cause we have assigned is invariable, but we have many reasons for suspecting that suppuration has always preceded this formation of fungus, or growth of granulation as we would call it. Even in the case in which Mr. Lawrence's friend performed the operation, there is great reason to believe, that slight suppuration, attended as it always is, where the curative process is regular, by surrounding adhesive inflammation, may have taken place in the substance of, or immediately under the tunica albuginea. However, we are not disposed to lessen the merit of Mr. Lawrence's communication. The appearance of the part, under these circumstances, will be always formidable to young practitioners, particularly so, if he sees it before the general intumescence and pain have entirely ceased. The description was therefore much wanted, and it is here given in an intelligible and satisfactory manner.

ARTICLE 2. — *Report of the General Hospital near Nottingham.* By JAMES CLARKE, M. D. one of the Physicians to the Charity.

THIS is the third paper from the same quarter. It contains useful practical remarks, but most readers will think them too much detailed.

ARTICLE 3. — *Memoire sur la Mortification de la Cornée.* Par J. P. MAUNOIR, de Genêve. Translated and communicated by Dr. de Roches.

THIS article contains an useful practical remark on the delicate operation of extracting the Cataract. It is the author's opinion, that those unsuccessful operations, which are followed with considerable discharge of water and with pain, which last subsides altogether, are not the effect of matter formed, but of high inflammation terminating in gangrene of the cornea. The cause of the gangrene he conceives to be, that the section is larger than necessary for the escape of the cataract. Hence, the circulation in the cornea, scantily supplied with vessels in order to preserve its transparency, is so much impeded, that no adhesion of the cut surface can be procured, and the part dies in consequence. This is extremely rational; we see no objection to the opinion, excepting the pain which attends the process, and this may be accounted for by inflammation in the globe and eye-lids.

The translation of this paper, though we doubt not very correct, yet is not without Gallicisms, which render it obscure in some parts. The Latin quotations are also incorrectly printed, an inconvenience extremely difficult to prevent.

ARTICLE 4. — *Observations on the Nature of Inflammation, and its Connexion with Fever; communicated in a Letter to Dr. Andrew Duncan, junior.* By Dr. A. PHILIPS WILSON.

THIS paper contains several experiments on living animals, to show, that in inflammation the action of the capillary vessels is lessened, whilst the action of the larger blood vessels is increased; but we shall endeavour to do justice to Dr. Wilson by following him seriatim. He first remarks, that mere obstruction of the minuter vessels is insufficient to produce increased action in the larger, because the obstructed blood is easily carried off by the anastomosing branches, or the vessels may resist the distending force. The Doctor passed a hot wire through the web of a frog's foot. This of course, produced a shrivelling round the part. No fluid escaped; the blood was therefore obstructed, but no sign of inflammation followed.

Respecting the general opinion that the circulation is more rapid in an inflamed part, it is first shown by experiment on a living frog, that in proportion as the action of the vessels increased, the part became paler, which is accounted for by their greater constriction. Here, therefore, was increased action without the true marks of inflammation. In another instance in which inflammation flammation actually existed, the vessels were larger and fuller, but the motion of the blood was more languid, and in some parts where the inflammation was greatest, the circulation had ceased altogether. The smaller vessels, which in a healthy state are impervious to the red particles of the blood, were injected.

"Whilst," says Dr. Wilson, "I was viewing the inflamed web, it occurred, that if I could succeed in stimulating its vessels to action, and thus remove the inflammation, it would afford an additional proof of the inflammation depending on the debility of the vessels.

"With this view I wetted the inflamed web with distilled spirits, at the same time throwing upon it the concentrated rays of the sun from the speculum of the microscope. The blood in all the vessels, except in those of the most inflamed part, began to move with greater velocity, and in proportion as this took place, the diameters of the vessels were diminished, the redness became evidently less remarkable, and the interstices of the vessels less opaque.

"After I had despaired of restoring action to the most inflamed part, I saw the blood begin to move slowly in a vessel which ran directly through the middle of it. It soon acquired a considerable velocity; and, on taking a superficial view of the part through the microscope, the course of this vessel appeared like a streak of a lighter colour through the middle of the inflamed part.

"As I had not observed the inflammation from the commencement in this experiment, I repeated it with the assistance of the Reverend Mr. Boraston, on a small fish (the lampern.)

"We found that exposure to the air produces a degree of inflammation, evident to the naked eye, in the fins and tail of this fish. On viewing the former through the microscope, we observed the circulation become more languid, and the vessels enlarge as the inflammation came on. The motion of the blood in the most inflamed vessels at length ceased altogether.

"By gentle friction, and applying distilled spirits, we repeatedly succeeded in accelerating, and even renewing the motion of the blood; and, in proportion to the velocity of the circulation, the vessels became evidently paler, the deeper red returning as the circulation became more languid."

This experiment was repeated on the fin of a fish with nearly the same result.

To see how far the same would happen, in what are called warm blooded animals, a protruded part of the mesentery of a rabbit was brought within the field of a microscope, and irritated by the point of the forceps; the observation being made by a Gentleman unacquainted with the theory, whilst the Doctor conducted the experiment.

"The larger arteries and veins were too opaque to admit of my distinguishing the motion of the blood; but in the small vessels, vessels, which were more transparent, the circulation was easily observable; and I perceived the globules of the blood moving along with great rapidity, but not in sufficient quantity to give a red colour to the vessels.

"After a few minutes exposure to the air, the vessels became visibly enlarged, and in some parts assumed a reddish colour, while the velocity of the blood was proportionably diminished.

"As soon as a part of the mesentery, which lay within the field of observation, and appeared almost colourless, was irritated with a point of a small pair of forceps, a red spot appeared. In a few seconds it increased in size, the adjacent parts of the vessels were distended, and the current of blood becoming less rapid, was for some distance slightly tinged with a red colour. This enlargement of the vessels gradually extended; the circulation at this time was extremely languid, and at length was not discoverable at all."

Such then, continues the author, appears to be the state of a part under inflammation; the capillary vessels distended and debilitated; the larger arteries excited to increased action.

The increased heat with which inflamed parts are attended, must, it is added, arise from the blood parting with m re caloric; but as part of this blood remains without again making its passage through the lungs, it is less in a condition to evolve caloric, than the blood of a healthy part. Mr. Hunter found by experiment, that the increased heat of inflamed parts is not more than 1 in 97 or 98; and, as by our author's experiments, the increased quantity of blood is considerably more than doubled, it follows, that less than half the above quantity of heat is evolved in a given space under inflammation from the same quantity of blood.

The increased pulsation of the larger arteries is imputed, as may be expected, to the *vis a tergo* endeavouring to expel the forward, the tardy, or stagnant blood through the capillaries. If this *vis a tergo* is insufficient, the consequence is, that the circulation ceases altogether and mortification follows.

The *vis a tergo*, we are told, is not only increased by the obstruction to be overcome, but the secretions of the inflamed part being interrupted, that portion of blood which would have been used for these secretions remains, and adds further to the stimulus on the vessels, which is gradually increased and extended backward to the heart itself.

Such are the outlines of Dr. Wilson's theory of inflammation, which, if rightly understood, will readily illustrate his theory of fever

"On reviewing," says he, "the phenomena of fever, we shall find them altogether analogous to those of inflammation, and that the experiments which have been related can be applied with the same success to explain them. The symptoms which constitute inflammation are increased heat, redness and swelling, whether attended attended by pain or not. In the commencement of the hot stage of fever, particularly in strong habits, the whole surface is affected with increased heat, redness, and swelling. Here, then, we know from direct experiment, that the capillaries are debilitated. The more active symptoms of inflammation do not shew themselves, because the whole capillaries being debilitated, the distending bears a small proportion to the resisting force; whereas, in inflammation, the debility of the capillaries being very partial, the resisting bears a small proportion to the distending force.

"The hot stage of fever, then, must be regarded as a state of general inflammation, (See my Essay on the Nature of Fever, pp. 186, 187); and the cold stage which precedes it, differs from the hot only in this, that the general debility of the capillaries exists with a debility of the heart and larger vessels, instead of an increased action of these organs, which always, in a short time, supervenes. For as, in inflammation, we observe, that as the capillaries of the part become debilitated, the larger vessels in their neighbourhood are excited to increased action; so, in fever, where the whole capillaries are debilitated, all the larger vessels are, for the same reason, excited to increased action.

"It has been said, that there is no proof of the excrementitious parts of the blood, when retained, proving a stimulus to the heart and larger vessels. In reply to this, it may be observed, that in all cases where they are retained, we observe the increased action of the heart and larger vessels; and as this increased action is evidently the means employed to restore the due action of the capillaries, there can be little doubt that the retained excreta are calculated to excite it; and we find that it always continues till they are expelled, or the larger vessels lose their tone. But whether this be admitted or not, the increased volume of blood in the larger vessels, which is the necessary consequence of the impediment opposed to its passage through the capillaries, must apply to them an increased stimulus, and consequently produce the phenomena which constitute the hot stage of fever.

"As, on the one hand, a debilitated state of the capillaries always produces increased action of the heart and larger vessels, so on the other, increased action of them, if long continued, never fails, from whatever cause it arises, to occasion debility in the capillaries. In my Essay on the Nature of Fever, I had occasion to observe, that when this disease terminates favourably, a degree of general debility remains after its symptoms disappear, but the action of the capillaries is so far restored, that it is in due proportion to the remaining vigour of the heart and larger vessels. In short, what now takes place throughout the system is analagous to what happens during resolution in an inflamed part. The extreme vessels are capable of effecting the necessary changes in the fluids supplied to them by the larger vessels. Thus, even the general debility which succeeds fever is a wise provision of nature; and and thus it is, that the debility of the capillaries, and along with it, the fever, is often renewed for some time after it has ceased, by too full a diet, exercise, or any other cause that increases too much the force of the heart and larger vessels, and thus throws on the capillaries a larger quantity of the fluids than on the first return of their vigour they can easily bear. A full meal, under these circumstances, is often succeeded by a strong pulse and a dry skin."

By the analysis we have made, and the extracts transcribed, it will not be difficult to see the object of Dr. Wilson, which we were formerly obliged to confess was beyond our reach. It is not necessary that we should offer any opinion of our own, because, we are informed, that in an edition of his Treatise on Febrile Diseases, which he is now preparing for the press, the subject will be entered into more at large. Whenever that edition appears, we shall have the whole of the question before us, and then will be the time to discuss it with that candour we always wish to shew every author.

Article 5.—*An Instance of Condensed Lungs, related with a View of enquiring whether or not this Morbid State ought to be considered as of a different Kind from Inflammation or other Diseases already distinguished.*

The subject of this case was a lady in the sixth month of her pregnancy, seized with pains in her chest, dry cough, short breathing and a shivering fit. The pain increased with the other symptoms till morning; the patient was bled; the same was repeated in the evening; the pulse small and quick, but the heart making violent efforts to contract. On the 4th day the bleeding was repeated. On the 5th, a violent pain in the head absorbed all other sensations; this induced a free application of leeches for immediate relief; with what success we are not informed, but that the patient seemed in a dying condition; and as a temporary relief, cupping with the scarifyer was directed for the chest. She miscarried, and died.

The following is the account of the dissection, with the author's remarks.

" *Dissection.*—On Thursday, April 14th, on opening the thorax, the lungs appeared not at all collapsed, but were firm like liver or spleen; the right lung was more consolidated than the left. A little semi-transparent, seemingly coagulated lymph, was found adhering to the pleura of the right side; and about eight ounces of water were taken out of the cavities of the chest, especially from the right side. The lungs being cut through in various parts and directions, the air vessels seemed obliterated, so that a very small part only of them floated on water. The right lung throughout was so dense as to sink in water. The colour in the interior was like the surface. There were no tubercles, nor vomicæ, nor thickened membranes, nor swelled glands; but the most remarkable appearance was the effusion of a turbid fluid into the trachea, so as to fill it, and which, no doubt, occasioned suffocation. This turbid fluid consisted of *serum*, *water*, *slime*, or *mucus*, and some *coagulated masses* of lymph.

"There was nothing unusual about the heart, and the foramen ovale was not, (as in many cases of diseased lungs) opened. In other parts of the body nothing morbid was seen.

"*Remarks.*—From the propensity to generalize, I know that the above described disease and dissection would be referred to *inflammation.* But on consideration of the phenomena, the characters of inflammation will be found to have been absent. It is true, that effusion of watery fluids, and of coagulated lymph, and condensation, are effects frequently of inflammation; but then these effects are attended by decisive and peculiar signs of inflammation, viz. redness, thickening or enlargement, adhesions, pus, and, previously to death, by symptoms of general, or at least local inflammatory action. In the preceding case, we have neither the symptoms of pleurisy, nor of peripneumony, nor of pneumonia. The pain of the side was supposed more like a spasmodic, than an inflammatory one. Effusion of water, and secretion of slime, are not the effects peculiarly of inflammation, but of various kinds of irritation and obstruction to the circulating fluids. It seems against experience and analogy, that for three or four days, inflammation of every part of both lungs should exist, without the symptoms of that disease, and yet sufficient to occasion an universally condensed state of them. Obstruction to the passage of the blood accounts for the effused fluid into the trachea, and into the cavities of the thorax; but what occasioned the consolidated state of the lungs? The consolidated state appears to have been the original disease, not the effect of some other disease; and its occurrence to so great an extent in three or four days, has not, I believe, been seen before. The absence of orthopnœa, of buffy blood, of hard pulse, of white tongue, of expectoration, and but little of cough, or any other particular, distinguish the present disorder from other well known thoracic affections. The rapidity of its progress will not escape attention. The dyspnœa was a peculiar one. The violent attack of peculiar excruciating pains of the head is also remarkable. The state of pregnancy is, perhaps, to be regarded merely as a casual coincidence, and not as peculiarly connected with the disease. Death immediately by secretion into the trachea, I believe, has frequently been overlooked; it often occurs in infants, and accounts for unexpected deaths."

As no name is appended to this communication, we shall not be accused of want of candour in offering a few remarks on it. The author conceives the disease no way connected with pregnancy. Had nothing appeared but the effusion into the trachea, we might have been of the same opinion; but it is certain, that during pregnancy, a very extraordinary quantity of blood is often made; witness those prodigious hæmorrhages which at another time no woman could support; witness the relief which bleeding affords under most complaints complaints during pregnancy; witness the great loss of food by perpetual vomiting, under which females suffer much less than at any other period. This increased quantity of blood seemed determined to the lungs, and at one time, to the head, (whether the latter was examined we are not informed.) In this unnatural state of the lungs, the cells must either burst, and the blood be effused into the air cells, or adhesive inflammation must take place to support the parts. Considering the texture of the lungs, it is obvious that if the disease continued, the adhesion must continue, till by degrees the office of the lungs would be destroyed. If, in the mean while, lymph should be extravasated into the distant branches of the bronchiæ, it would find its way to the trachea, but could not be coughed up from the condition of the lungs. All this we offer as conjecture, and should a similar case occur, if we may judge by what we have witnessed ourselves, the first bleedings, and the repetition of them, should not be measured by ounces, but by the relief experienced, and the indication of the returning symptoms.

ARTICLE 6.—*Instance of an Endemic Cynanche Parotidea, on board his Majesty's Ship Ardent, on her Passage to Monte Video.* By ANDREW NOBLE, Surgeon of the Ardent.

THIS is a very interesting paper, showing the occurrence of an endemic, which, in the course of seventeen days, attacked twelve of the crew, without any cause to which it could be attributed, either of weather or contagion. We are informed the ship had just entered the trades in the month of November; but the author should have added how long he left England, or whether he had touched at Madeira, or any other Island in his way. This is an omission which is easily remedied, and which, we doubt not, Mr. Noble will communicate, to make his paper as complete as possible. In some cases, the brain sympathized to such a degree, thst it was found necessary to repeat the bleeding to a very considerable quantity; the testicles, and after them the brain, took up the action in succession with so much violence, that it was found necessary to repeat bleeding and other evacuations to very great extent. This succeeded in all, and with a rapidity proportioned to the boldness of the practice.

ARTICLE 7.—*Case of Tic Douloureux cured by Calomel and Opium.* Communicated by JAMES CORKINDALE, M. D. Member of the Royal Society of Edinburgh, and one of the Surgeons of the Royal Infirmary, Glasgow.

THOUGH this composition has been often tried without success, yet in so disheartening a disease, it is a great relief to meet any cases which have yielded to this or any other remedy.

ARTICLE 8.—*Observations on the Effect of Purgative Medicines.* By J. CHEYNE, M. D.

PURGATIVE medicines are now become such a hobby-horse, that a stranger, to read the frequent mention of them among the medical writers of this time, might fancy they were unknown or unused, till Dr. Hamilton published his useful treatise on that subject. The case ease with which Dr. Cheyne introduces his observations, is very well worth recording; but the greater part of his subsequent remarks might have been omitted. As we have no reason to suppose that any of our readers at present are ignorant of the propriety of attending to alvine discharges in all chronic diseases, we shall decline any further notice of this part of the paper. By some quotations produced, it would appear as if some judicious, and even celebrated practitioners, were not sufficiently aware of the impropriety of strong stimulating purges in cases of inflamed bowels. The author's observations, in this respect, may be useful, but he is not sufficiently minute in describing the symptoms of inflammation. Where the pulse is small, the pain violent, the tension and tenderness considerable, the free use of the lancet, and, perhaps, of topical bleeding, is absolutely necessary: nor can we expect any advantage from purges, however strong, before we have subdued the inflammatory symptoms.

ARTICLE. 9.—*Reply to Dr Bostock's Remarks on Mr. Ellis's Treatise on Respiration.* By Mr. DANIEL ELLIS.

NOTHING can give us greater satisfaction, than a controversy carried on like the present. We have already shown our high opinion of Mr. Ellis's labours, by the early notice we took of it in our critical analysis; we have not been less attentive to Dr. Bostock's reply. Each is replete with good sense and good manners, and we hope the correspondence will be kept up with the same zeal and temper on both sides. We shall for the present, look on as impartial spectators, till something new appears, or till we perceive something new to offer our readers.

The Inquirer occupies his corner; and it is not a little curious to see Dr. Ferriar accused of the same plagiarism as he has effectually fastened on Sterne. It appears that Lorry, in the year 1784, published a Tract on the conversion of diseases. Some passages are brought, to show the similarity of the two. The author does not seem quite aware to how much advantage Dr. Ferriar appears in the comparison. It is, indeed, afterwards said, that "in treating of a complicated series of morbid actions, there is great danger of being bewildered in the maze of chimerical notions, and this the more especially if the author sets up for a system-maker. Something of this kind, we apprehend, has happened to those who have examined the conversion of diseases before Dr. Ferriar. He has pointed out the true method of inquiry, he has added a good example to a judicious precept, and endeavoured to recal physicians to that line of study by which Hippocrates and Sydenham enjoy a sort of immortality upon earth, and at this day read lectures to all the world."

Thus, after a spice of abuse, the Doctor is brought forward in most excellent company. But poor John Hunter, in the passage immediately before us, is brought into sad disgrace, and there left without an apology.

"In one of his chapters, where he speaks of the changes which the natural powers of the body bring about, and traces the ineffectual tual influence of certain actions, he states most distinctly the observation which has been attributed to Mr. John Hunter, and held up by some of his over-zealous admirers as his theory of the incompatibility of similar morbid actions in the living body at the same time, which, in truth, is nothing more than a recital of facts in abstract terms: 'Id enim omnis semper medicus ante oculos habere debet, quod observatio demonstravit ab ipsius Hippocratis ævo constantissima; *naturam salubriter ad duo objecta non posse attendere* et in ipsis occupari, sed è duobus alterum ab altero hebetari et quasi excludi.'

Now, we should be glad to know in what passage Mr. Hunter ever says that two *similar* morbid actions at the same are incompatible. We have frequently heard his disciples assert, that two local diseased actions cannot be carried on in the same place, nor two diseased actions in the same constitution. But the similarity of the actions would supersede the whole theory, which only rests on the difference: and it now appears, that small-pox and cow-pox, tho' both constitutional, can go on at the same time, on account of their similarity. But it is hardly conceivable, how the writer should have overlooked the difference between the two writers. J. Hunter speaks of diseased actions, one of which will be suspended, whilst the other is proceeding. Lorry speaks of actions tending to health, one of which, he says, will be torpid, or altogether excluded by the other. This is a matter of very little consequence, as Mr. Hunter delivered his opinion before Lorry, but it is as just that Lorry should be excused from an unjust charge of plagiarism as Hunter or Dr. Ferriar.

---

*Essays on the Morbid Anatomy of the Human Eye.* By James Wardrop, Fellow of the Royal College of Surgeons, of the Royal Medical and Chirurgical Societies, and one of the Surgeons of the Public Dispensary of Edinburgh. Illustrated with Plates; large 8vo. Edinburgh, 1808.

In a Preface, the Author observes, that a work professedly on the Morbid Anatomy of the Eye, is hitherto a desideratum: the Continental writers, who have principally engaged the public attention, having done but little towards illustrating their remarks by engravings. He acknowledges the information he has derived from these writers, and from the practitioners of his acquaintance, and concludes by informing us, that should the present specimen be well received, it is his intention to prosecute his plan, by considering the remaining diseases of the eye and its appendages, with the treatment which such diseases require.

Some preliminary observations follow; that all animals are composed of organs, which, under the influence of the vital principle, produce those wonderful phenomena that distinguish living organized bodies; that a knowledge of organs, is one of the objects of anatomy; that on it the surgeon builds all his theories; and and that it guides his hand in every operation. This, however, is only to be considered the first step.

"When," says our author, "the anatomist divided the body into regions and districts, and shaped his inquiries to suit his unnatural divisions, every organ appeared insulated and detached, the most minute parts might have been discovered and described, but their mutual connections and sympathies were unknown. Anatomy and physiology were then disjoined; the former was imperfect, and the latter could scarcely be said to exist.

"A more minute and philosophical examination of the structure and properties of the different organs, led the way to a knowledge of some of their functions, and pointed out the principles which should regulate the investigations of every rational physiologist. Haller was among the first to avail himself of the advantages of this plan. It conducted him to all his important discoveries; and it has determined the progress of every scientific inquirer since his time. To it we are indebted for almost every improvement that has been made in this branch of science; and it is the only method by which we can hope still further to augment our knowledge. In pursuing this track, the labours of modern anatomists have been well rewarded. They have freed physiology and pathology from the nonsensical conjectures by which they were so long debased, so that they now begin to assume their rank among the sciences, and in some cases to afford a a safe guide to the medical practitioner."

That Haller did much towards correcting many anatomical errors and completing some researches cannot be doubted; it is, however, still doubtful whether, he has done much towards improving Physiology.—But we shall continue to transcribe.

"No one in our day has exerted himself more successfully in this field than the late celebrated Bichât. His *Anatomie Generale* is one of the most remarkable productions that has ever been produced in medical science. It has unfolded a path of investigation which was scarcely ever trodden before, and laid the foundation of a new anatomy and a new physiology. I cannot pretend here to do justice to the merits of this work, nor to give a correct view of the facts and reasonings by which his doctrines are supported. They are as numerous and various as are the parts and functions of the living body. But, as I propose, in examining the pathological anatomy of the eye, to adopt some of the principles which he has established, the following observations are necessary, in order to explain the purport and tendency of the classification which I mean to adopt.

"Most of the organs of our body are made up of a variety of elementary parts, or textures, each of which, in whatever situation it is found, affords uniformly the same physical properties. These are the elementary parts, which, by the diversity of their combinations, produce all the modifications of structure and functions which the different organs of animals exhibit. The study of

'these elementary parts, independent of the organs which they concur to form, is the object of *general* anatomy.

"This method of considering organized bodies, is not an unnatural abstraction, nor a speculative refinement. It arises from the essential nature of their constitution, and it accords with every phenomenon with which we are acquainted. We may trace it in the observations of many of the older anatomists; and it may be considered as the basis of some of the most ingenious physiological theories of the late celebrated Mr. Hunter. Although, therefore, at first sight, it may have the appearance of being arbitrary and artificial, it is nevertheless, I am persuaded, founded on the most approved principles of philosophical investigation. A knowledge of the qualities of the different parts of which our organs are composed, must afford the surest means of acquiring information concerning the functions of these organs, and of becoming acquainted with the changes which they undergo in disease. On these principles Bichât has founded his anatomical system. To numberless experiments upon living animals, he has added all the information which could be acquired by dissection. He employed chemical re-agents to supply the deficiences of the scalpel, and examined with minuteness all the varieties of morbid structure. By these means he endeavoured to fix the characters of the elementary textures, and then proceeded to investigate their combinations, as they are naturally presented to us in the different organs.

"Of these textures, he has enumerated twenty-one, all of which he has shewn to be differently organized; and hence he proves the dissimilarity of their properties, both in health and in disease. This is the ground-work of the whole fabric, and to it we must ultimateley recur in every attempt, to account either for the natural or morbid appearances which are to be met with among organized beings."

"I mean not," continues Mr. Wardrop, "to enter more minutely on the consideration of elementary textures." We should be extremely glad to learn what *elementary textures* mean, or why they are to be entered upon at all, if not to introduce some practical remarks. If the meaning was to shew that parts differently organized exhibit different phenomena when under inflammation, it would have been sufficient to trace some of those laws which Mr. Hunter has pointed out, in explaining the various forms of inflammation. We are indeed slightly referred to him and to Dr. Carmichael Smith's confused attempt at arranging the different species of inflammation; but BICHAT is the source of all our Author's speculations. Bichat has informed us, that mucous membranes, whether in the nose, the intestines, the vagina, the bladder, or in whatever part, are affected in a similar manner. It is much to be regretted, that Mr. Hunter's divisions of different kinds of inflammation, according to the nature of the parts affected, are not better known, that we might wonder less at these astonishing discoveries of M. Bichât and other foreigners.

After

After all, it may be doubted, whether, in a practical work, the inquiry is as important as it is interesting and curious.

"The principles which I have stated," continues our author, "account admirably well for the propagation of some affections, and for some of the sympathies which subsist between different parts of the body; but there are other disorders which advance in a different manner. In some diseases which are termed chronic, for example, the whole structure of an organ becomes gradually altered, although the primary affection was confined to one of its component textures. This is often to be observed in cancer, scrofula, lues venerea, &c. When cancer attacks the mamma, it is, at its commencement, generally confined to a small portion of that organ, but if allowed to proceed, it ultimately involves the whole glandular, cellular, and cutaneous textures, in one common mass of disease."

It is far from our wish to undervalue the labours of those inquirers, who trace Nature in all her operations of disease, as well as health. But, we wish much, that every work should be *simplex et unum.* Mr. Wardrop cannot expect that these hints, for they are nothing more, can assist his readers in prosecuting such delicate enquiries, nor does the nature of his work require it. In a practical view, it is sufficient that he points out the symptoms of inflammation in the eye in its early stage. In the the progress, if one part is so much injured, that it cannot recover itself without chirurgical assistance, it is highly necessary to give us a most correct view of the parts, and of the mode of relief, whether by operation, or the application of other means. By this, we wish our readers to understand, that without entering into the question concerning elements or textures, we cannot help considering the whole of Mr. Wardrop's "preliminary observations," as altogether unnecessary or useless from their brevity.—We shall therefore proceed at once to the Essays.

This part of the work is confined entirely to the diseases of the cornea, including the conjunctiva, and the membrane which lines the inside of the cornea, and also some general remarks on the sclerotic coat. The author very justly observes, that the substance or texture of the cornea, is different from any other membrane with which we are acquainted, consisting of perfectly transparent lamellæ; the sclerotica he defines a fibrous membrane, inasmuch as its fibrous texture may be traced. The conjunctiva is with more propriety called a mucous, and the internal surface forming part of the cavities of the anterior chamber of the eye, a serous membrane.

The first diseased action treated of is inflammation of the cornea. After remarking the probability, that in such cases, all the various textures of which the cornea is composed may be effected; the author proposes, for the sake of greater accuracy, to consider the appearances of each under inflammation.

The inflammation of the conjunctiva covering the cornea is first

 described.

described. It is well distinguished from the speck of the cornea, and also from the pustule, which is formed by the membrane itself. The progress of the inflammation is accurately traced, till its termination in resolution, or in that new substance called pterygium* or pustule.

Inflammation of the proper or lamellated substance of the cornea, is next considered, and described with much accuracy through its whole progress to resolution, or its termination in suppuration or speck. The last, the author observes, "remains, and continues to be nourished by a number of red vessels." In this instance, we think Mr. Wardrop less accurate than we should have expected. Specks are sometimes formed by a mere adhesion of the various lamella in which we perceive no red vessels, yet the opacity remains. At other times, a mere effusion of lymph on the surface, obscures the proper transparency of the membrane. This last is, however, frequently absorbed, though slowly.

Three chapters follow, in which Pterygium excrescences, and pustules of the cornea, are more minutely described, and well illustrated with coloured engravings.—A suggestion is added, and strengthened by Professor Himly of Brunswick, that small pustules on the cornea are analogous to aphthæ on the fauces and alimentary canal. If they are really attended with those general symptoms described by our author, and relieved principally by attention to the constitution, there seems much propriety in the suggestion. Our own experience does not authorize us to give any opinion on the subject.

A chapter follows, on the abscess of the cornea and the anterior chamber of the eye. There is something obscure in the latter part. We perfectly agree with our author, that mucous membranes, in some stages of inflammation, secrete matter; or, as Mr. Hunter calls it, take on the suppurative inflammation; but this cannot be called abscess. The collection of authorities is, however, so curious, that we shall not scruple to transcribe a part of the chapter, to show the diligence of the author in collecting useful materials for his work.

"Schiegel mentions a case of abscess of the anterior chamber, in which the pores of the cornea were so open that the matter oozed out in the form of threads. The following are his words, 'By the application of the decoction in a case of hypopion, the matter discharged itself in an uncommon manner. Whilst using it, the fine pores of the cornea were opened, and the matter oozed out in the form of fine threads. On the second day the distended cornea was considerably flatter, the oozing out of the matter continued without interruption, and in four days, nearly two drachms of matter had passed through the pores.'

"These abscesses are commonly the effect of violent ophthalmia, occasioned by blows, or injuries of the eye-ball. Richter, however

* See our Review of Scarpa.

ever, remarks, that purulent matter sometimes collects in venereal and scrophulous patients, without any preceding inflammatory symptoms. Dr. Rutherford mentioned to me the case of a woman who was under his care in the Royal Infirmary of Edinburgh, who had a very considerable collection of matter in the anterior chamber, accompanied with very little or no inflammation. The matter altered its form and place, according to the position of the head, and, during the day, the agitation of the body, produced from walking, mixed the matter with the aqueous humour, and rendered the whole anterior chamber turbid. Janin relates a very curious case, where there was not only the absence of the inflammatory symptoms, but where the disease recurred periodically. 'About the beginning of the month of March, 1757,' says he, 'Peter Valis consulted me about a periodical blindness with which he had been affected for twelve months, during the first fifteen days of every month; and, after that time had elapsed, his eyes were restored to their natural state. I examined the organ, in order to ascertain the cause of that singular kind of blindness, and I observed that the anterior chamber of both eyes was filled with a yellow-coloured matter, so thick, as neither to allow the colour of the iris, nor the state of the pupil to be seen through it. The most remarkable circumstance in this case was, that the conjunctiva was very little inflamed, and the eye not painful.

"Richter saw a man who was blind every morning, and it was always remarked that, while the paroxysm lasted, the aqueous humour was quite turbid.

"It has been a subject of dispute, to account for the source of the matter which is formed in hypopion. It appears most probable, that as there is no ulcerated surface from which it can be derived, it is the produce of some secreting organ. There are numerous examples of the natural secretion of surfaces being altered by diseases. It is very remarkable in the mucous membranes, and in those of the serous class, to which the membrane which contains and secretes the aqueous humour belongs. When they are inflamed, it is a very common morbid appearance to observe their surfaces covered with a matter, varying from the consistence of a thick coagulated lymph to that of a thin yellow puriform fluid."

A chapter follows, on one of the sequelæ of inflammation, or abscess, viz. ulcers of the cornea. They are also traced from the application of corrosive substances and other causes. The mode of future cicatrization is accurately traced. Wounds of the cornea are next considered, and afterwards some very useful remarks follow on extraneous bodies adhering to the globe of the eye, or to different parts of it.

The chapter on the ossification of the cornea might have been enlarged; but it must be admitted, that very little practical information could have been derived from tracing a diseased action, which we have no means of altering.

The ninth chapter, on the Speck of the Cornea, is long, and arranged ranged with care, in proportion to the subject. The manner in which the different varieties are illustrated by engravings, prevents our doing justice by any extract or analysis. We must, therefore, content ourselves with a bare enumeration, leaving the reader to examine the work for an accurate description. Four distinct varieties are very well marked, and several less striking appearances accurately described. They are all illustrated by engravings. The following paragraph is, however, to us, by no means satisfactory; not only because we have never seen in a young subject any thing like what is very properly called *arcus senilis*, but because the drawings, to which we are referred, give by no means a satisfactory idea of that state of the eye.

"In some cases the opacity, instead of being formed towards the central part of the cornea, or at some distance from its circumference, begins at the place of the junction of the cornea and sclerotic coat, and gradually extends towards the centre of the cornea, forming an opaque ring around its circumference. Most authors have described this as an appearance only to be remarked in the eyes of old people, and have given it the name of *arcus senilis*. But I have observed it at all periods of life, and it may be seen in several of the drawings which were taken from young subjects. It generally appears like a bluish ring at the edge of the cornea, running round the whole circumference. It is almost universally to be found in the eyes of old people, and it increases in breadth as the person advances in life; but it is never attended with any impediment or inconvenience to vision."

This chapter concludes with some remarks on the formation of specks. It can hardly be questioned, that inflammation must be the cause of specks, whatever the cause of inflammation may have been. But we cannot help feeling distressed, when we see lues venerea and scrofula so perpetually brought forward as the cause of various complaints. The most accurate and rational writers are unwilling to admit that lues venerea ever occasions ulceration in the eye, and if it did, the progress of this disease is so slow, that it might always be arrested, so as to leave the eye in a perfect state. But we have never met with an author who could describe the venereal state of the eye, or ascertain when it may, like all other diseases from that source, be with certainty cured by mercury. Instead of the term scrofulous, we conceive the chronic inflammation much less exceptionable, because a more intelligible expression. The following is the author's account of the appearances of *specks* on dissection.

"When the cornea is examined after death, no change of structure can be observed in those cases where there had been a mere cloudiness, or general opacity during life; for even before death, more especially if it is slow and lingering, the fluid which, in the natural state, is deposited between the lamellæ of the cornea, exudes, forming an obscure layer over its anterior surface, and the aqueous humour oozes out, giving the cornea an unequal puckered appearance.

appearance. Indeed, it is from this change in the eye that approaching death is often foreseen; for whenever the cornea begins to collapse, and becomes turbid, the eye loses all its lustre and intelligence, and gives that awful expression to the whole countenance, which has been called *facies hypocratica*.

"When the cornea has been much more opaque, no other change is to be perceived after death, than a diminution of the transparency, either of the external lamellæ, or of the whole substance of the cornea. I have had many opportunities, in the living body, of taking off layers of very opaque specks, and I have never been able to observe any other change, except that, in some of those which have been of long duration, the cornea had acquired a degree of hardness much greater than that of a sound cornea.

"In most cases, too, a speck bleeds when a piece of it is removed in the living body; and I have observed this happen, even when no red vessels passing into it could be detected by the naked eye. An incision made in the healthy cornea, gives little or no uneasiness, but, when the portion of a speck is removed, it often excites acute pain."

We have extracted this passage, first, because, though we passed it over before, yet we conceive this *cloudiness* of the eye, as it is here very properly called, should not be confounded with the speck; and, next, because in the instances in which we have seen the thickened cornea cut very freely, when the symptoms of inflammation were not present, the patient has never complained of much pain, perhaps of none, how great soever his fears may seem.

A chapter follows, on the staphyloma, or the prominent tumour; another on alterations in the figure of the cornea; and, lastly, one on the affusion of blood into the lamellæ of the cornea, and into the anterior chamber. At the close of the book, are seven beautiful engravings, six of them coloured, and each containing three figures, with copious explanations annexed.—Such is the nature of this work, which may clai [illegible], besides its intrinsic merit, a degree of novelty: for though some of the old French writers have given as numerous engravings of diseases in the eye, as of the attitudes in fencing, yet it would be great injustice to compare them with the performance before us. We sincerely hope the author will be encouraged to proceed; and if he is not displeased with the hints we have taken the liberty to offer, he will have ample opportunities of availing himself of them in the progress of his work.

